# Are Cyberchondria and Intolerance of Uncertainty Related to Smartphone Addiction?

**DOI:** 10.1007/s11469-023-01054-6

**Published:** 2023-05-03

**Authors:** Aleksandar Vujić, Marija Volarov, Milan Latas, Zsolt Demetrovics, Orsolya Kiraly, Attila Szabo

**Affiliations:** 1 Doctoral School of Psychology, Faculty of Education and Psychology, ELTE Eötvös Loránd University, Budapest, Hungary; 2 Institute of Psychology, ELTE Eötvös Loránd University, Budapest, Hungary; 3 Department of Psychology, Faculty of Sport and Psychology, Educons University, Novi Sad, Serbia; 4 Faculty of Medicine, University of Belgrade, Belgrade, Serbia; 5 Centre of Excellence in Responsible Gaming, University of Gibraltar, Gibraltar, Gibraltar; 6 Institute of Health Promotion and Sport Sciences, ELTE Eötvös Loránd University, Budapest, Hungary

**Keywords:** Behavioral addiction, Cyberchondria, Intolerance of uncertainty, Problematic smartphone Use, Smartphone addiction

## Abstract

Smartphones are a medium for performing online activities, and one such activity could be the compulsive online health information search — cyberchondria. This study aimed to test whether cyberchondria and intolerance of uncertainty (IU) positively predict smartphone addiction (SA), adjusted for age, gender, daily use duration, the reason for using smartphones, and symptoms of anxiety and depression. The sample consisted of 471 adults (55.2% women) from the general population without chronic diseases (*M*_age_ = 38.67). Regression analysis showed that IU was a positive predictor of SA (*β* = .17, *p* < .001), as well as cyberchondria (*β* = .14, *p* < .001), which had a unique contribution to the explanation of SA, relative to IU. Other significant predictors were average daily smartphone use and entertainment use, the latter being the strongest predictor in the model. These results revealed cyberchondria as a unique predictor of SA.

## Smartphone Addiction — Definition and Conceptual Dilemmas

Smartphone addiction (SA) is “…a compulsive pattern of smartphone usage which can result in negative consequences that impair the daily functioning of the user” (Busch & McCarthy, 2021, p. 2). In the current study, we use Griffiths’ (2005) components model of addiction as a framework. Based on this model, SA is “… a behavioral addiction including the core components of addictive behaviors, such as cognitive salience, loss of control, mood modification, tolerance, withdrawal, conflict, and relapse” (Billieux et al., 2015a, 2015b, 2015c, p. 157; Griffiths, 2005). However, SA is a controversial term among researchers because there is increasing criticism of pathologizing components of the modern lifestyle and labeling *problematic smartphone use* (PSU) as a *behavioral addiction* (Billieux et al., 2015a, 2015b, 2015c; Billieux et al., 2015a, 2015b, 2015c; Flayelle et al., 2022; Kardefelt-Winther, 2017; Panova & Carbonell, 2018). Although some novel results support the view that PSU/SA is a behavioral problem that has brain abnormality correlates (e.g., Zou et al., 2022), we agree that it is too early to consider this phenomenon a non-substance addiction, like gambling disorder. However, we decided to use the term SA to denote PSU for easier comparison with other studies. Smartphone addiction and PSU are commonly used in literature as synonyms (Busch & McCarthy, 2021), and SA can often be found in the names of the measurement tools that tend to measure PSU (Tossell et al., 2015). To sum up, even though we use the term SA in the current study, we refer only to a possible “risk” for addiction rather than a diagnosable behavioral addiction. This approach aligns with the idea that problematic use and addiction lie on the same continuum, where inappropriate use can turn into addictive use, similar to what has been suggested for problematic gambling and gambling addiction (Griffiths, 2016).

Despite their excellent functionality, smartphones are of limited use without an internet connection (Montag & Reuter, 2017). Therefore, there have been proposals to rename the SA construct as “internet use disorder, predominantly mobile,” to emphasize the problematic internet activities performed via smartphone (Montag et al., 2021). This term shows how problematic internet use (PIU), and SA/PSU are often difficult to distinguish. However, smartphones have distinctive features (such as portability, constant connectedness, and compact size) that can uniquely impact behaviors performed on the internet. Those smartphone features make people more susceptible to developing an “addictive” behavior (see Barnes et al., 2019; Panova & Carbonell, 2018). Finally, it is worth mentioning that when researching behaviors related to PSU or the internet, regardless of the terminology, the focus should not be solely on the frequency of use but on the activities a person performs on the internet and on the motives that mainly drive such behaviors (Griffiths & Szabo, 2014; Pontes et al., 2015).

## Smartphone Addiction and Mental Health Problems: Relationship and Causality

Smartphone addiction is related to anxiety, depression, perceived stress (Elhai et al., 2017a, 2017b), loneliness, social phobia (Bian & Leung, 2015), sleep quality (Stanković et al., 2021), and poorer academic performance (Hawi & Samaha, 2016). A newer study suggests that the relationship between general distress and PSU could be indirect, through expectations of smartphone use and metacognitive beliefs (Casale, et al., 2021). In addition, excessive smartphone use is related to negative physical health outcomes, such as craniocervical problems (Kee et al., 2016; Park et al., 2015) and somatization problems (Winkler et al., 2020). Establishing the causality between PSU and poor mental health outcomes is difficult because both directions seem possible. On the one hand, psychopathological problems can lead to excessive smartphone use (e.g., if a smartphone serves as a means of maladaptive coping such as distraction or excessive reassurance seeking). Therefore, the association between psychological problems and SA can be explained from a compensatory technology use perspective (Kardefelt-Winther, 2014). To be precise, excessive smartphone use can manifest in a maladaptive coping style, where a person tends to compensate for the underlying psychosocial problems by using a smartphone (Billieux et al., 2015a, 2015b, 2015c; Kardefelt-Winther, 2017; Panova & Carbonell, 2018).

On the other hand, highly increased technology use can cause various problems with mental health, such as prolonged stress, depression, and sleep disruption. These dysfunctions are due to exposure to stressful content or comparing with people on social media who seem much happier and more successful (Elhai et al., 2017a, 2017b; Vogel et al., 2015). Also, a reciprocal relationship between mental health problems and SA is possible. For example, individuals could use smartphones to alleviate negative moods or to avoid real-life problems (Elhai et al., 2017a, 2017b; Wang et al., 2015). This form of coping corresponds to the mood modification component of SA that reflects the change in mood (arousing or calming) that people experience as a direct consequence of using a smartphone (Csibi et al., 2021; Griffiths, 2005; Zhang et al., 2014). However, the increased use can lead to more anxious and/or depressive states because smartphone-related activities performed to diminish or avoid negative emotions can be seen as a form of experiential avoidance (see Elhai et al., 2017a, 2017b). While experiential avoidance as a coping strategy can produce positive short-term effects (captured by the mood modification component), they keep the person away from problem-focused, goal-oriented activities, which harms one’s mental health in the long run (Hayes et al., 2016).

## Can Cyberchondria Predict Smartphone Addiction?

Behaviors related to cyberchondria have become particularly relevant since the surfacing of COVID-19 (Varma et al., 2021). Indeed, 60 publications on the Web of Science in November 2022 discussed the relationship between cyberchondria and coronavirus. Cyberchondria relates to searching for health information online to decrease distress or anxiety (Starcevic & Berle, 2013). Still, it increases anxiety levels, leading to more online searches, which becomes difficult to stop (Starcevic & Berle, 2013). Therefore, cyberchondria is closely related to health anxiety, illness anxiety disorder, obsessive–compulsive disorder, and PIU (Starcevic, 2020; Starcevic et al., 2019). However, cyberchondria has recently been recognized as a distinct and clinically relevant construct — a compulsive syndrome-like behavior related to adverse psychosocial outcomes (Vismara et al., 2020). The Cyberchondria Severity Scale (CCS) recognizes four distinct facets: compulsion, reassurance, distress, and excessiveness (McElroy et al., 2019). Network analysis indicated that each dimension measured by CSS played an equally important role in conceptualizing the construct (Starcevic et al., 2019). However, the Short Cyberchondria Scale (SCS) is unidimensional; its items refer to what the authors believed to be the core component of cyberchondria characterized by excessive online health information search and the associated distress (Jokić-Begić et al., 2019).

Previous studies showed that cyberchondria is related to compulsive internet use (Khazaal et al., 2021), or what was called PIU in Starcevic et al. (2019), or internet addiction (Ivanova & Karabeliova, 2020). Compulsion and distress domains of cyberchondria were most strongly related to compulsive internet use, linked to online search for health information (Khazaal et al., 2021). Given the robust relationship between cyberchondria and PIU (Starcevic et al., 2019), it is plausible to assume a positive relationship between cyberchondria and smartphone addiction. A person who exhibits cyberchondria-related behaviors, i.e., compulsive and excessive online health information search, which is difficult to stop, will use a smartphone to access the information online to decrease anxiety. However, if the wanted reassurance was not achieved, a person would enter a “vicious circle,” as the nature of cyberchondria suggests, and they will end up with higher levels of anxiety than before the search, leading to even more reassurance seeking online (Schenkel et al., 2021; Starcevic & Berle, 2013). One reason for this would be the characteristics of the information on the internet, which can be unpredictable and unreliable (Starcevic & Berle, 2013; Starcevic et al., 2020), as well as information overload (Laato et al., 2020). In short, the need for repeated reassurance leads to excessive internet use (i.e., via smartphones), which can turn into excessive smartphone use and, ultimately, addictive use.

Unfortunately, only a few studies investigated the direct relationship between PSU/SA and cyberchondria (e.g.,Köse & Murat, 2021; Yam et al., 2021). However, a moderate-high correlation emerged between cyberchondria and PSU/SA, *r* = 0.60 (Köse & Murat, 2021). Also, cyberchondria was a moderating and mediating variable between PSU/SA and fear of COVID-19 (Yam et al., 2021).

It is plausible that smartphones can facilitate cyberchondria-related behaviors due to their portability and practicality. Additionally, a study showed that most online health searches (i.e., cyberchondria-related behaviors) are associated with internet use during the night-time (Kanganolli & Praveen, 2020), where it is likely that such online search is done via smartphone (for example, while lying in bed). Smartphones also allow the health-anxious person to search for health information while commuting to work or on the worksite and in other places where it would be impossible to use a desktop computer. Using a smartphone as a tool to access the internet to alleviate health-related anxiety and distress can lead to problematic use, which interferes with daily life activities and increases anxiety even more.

Our research explores the relationship between cyberchondria and SA in the healthy population (individuals without chronic diseases). This delimitation was applied because illness could foster a search for online health information and lead to cyberchondria. This link was especially considerable during the COVID-19 pandemic when this study was conducted (seeArsenakis et al., 2021; Vismara et al., 2021).

## Intolerance of Uncertainty and Its Relation to Smartphone Addiction

Intolerance of uncertainty (IU) is “an individual’s dispositional incapacity to endure the aversive response triggered by the perceived absence of the salient, key, or sufficient information, and sustained by the associated perception of uncertainty” (Carleton, 2016, p. 31). Intolerance of uncertainty includes behavioral (e.g., inhibition or excessive collecting of information), cognitive (e.g., irrational beliefs about uncertainty), and emotional (e.g., being upset and frustrated) reactions to uncertain situations (Bottesi et al., 2020; Freeston et al., 1994). Many studies suggested that IU is a transdiagnostic risk factor for anxiety and mood disorders (Mahoney & McEvoy, 2012; McEvoy & Mahoney, 2012). Although IU is typically seen as a correlate of internalizing mental disorders, there is evidence that IU is also related to the symptoms of externalizing psychopathology (e.g., Sadeh & Bredemeier, 2021). It seems that IU is correlated with worry, rumination, and compulsive behaviors, but also with substance (ab)use and other risky/impulsive behaviors (Carleton, 2016; Freeston et al., 1994; Kraemer et al., 2015; Mihić et al., 2014; Oglesby et al., 2014; Sadeh & Bredemeier, 2021). For example, according to Sadeh and Bredemeier (2021), individuals with higher IU were likelier to engage in risky behaviors to reduce or avoid distress rather than to experience a pleasure. Similarly, in other studies, IU was found to be related to coping motives for alcohol use (Kraemer et al., 2015; Oglesby et al., 2014).

Based on the results of one meta-analysis, the level of IU was positively related to the use of the internet and mobile phones (Carleton et al., 2019). Smartphones can serve as a “safety blanket” since they increase reassurance possibilities through constant connectivity, disabling an individual from learning to tolerate uncertainty (Carleton et al., 2019). The intensified reassurance actions can catalyze increased perceived uncertainty, which leads to elevated anxiety (see Carleton, et al., 2019). This path is similar to the mechanism where cyberchondria, as a reassurance-seeking behavior, brings only short-lived relief from distress but increases anxiety in the long run (Carleton et al., 2019; Norr et al., 2015; Starcevic & Berle, 2013). Negative reinforcement makes an individual more likely to persist in reassurance-seeking (Carleton, et al., 2019; Salkovskis, et al., 2003; Tang et al., 2007).

Based on the compensatory internet use theory (Kardefelt-Winther, 2014), individuals with higher intolerance of uncertainty may experience anxious feelings more frequently, making them excessively use the smartphone to cope with the anxiety. This phenomenon is similar to the idea that stems from previous findings that suggest people with increased IU tend to consume more alcohol to deal with distress (Rozgonjuk et al., 2019). However, this kind of coping does not necessarily need to be reflected in reassurance-seeking online but also in different forms of avoidant behaviors, that include activities performed via smartphone. Accordingly, IU was positively related to PSU/SA and non-social smartphone use (Rozgonjuk et al., 2019), which was named “process use” (van Deursen et al., 2015), “entertainment use” (Horwood & Anglim, 2019), or “hedonic use” (Vujić & Szabo, 2022). Non-social use also mediated the relationship between IU and PSU/SA (Rozgonjuk et al., 2019). In addition, IU predicted nomophobia, a construct closely related to SA, and it mediated the relationship between differentiating self and nomophobia (Ercengiz et al., 2020). Nonetheless, clear causal direction could not be determined. That is, it is not sure if increased use of smartphones (and the internet) leads to higher anxiety through perceived uncertainty or if increased IU makes an individual prone to excessive smartphone/internet use and ultimately to SA.

Moreover, IU was proposed as a transdiagnostic risk factor underpinning various mental health problems (Mahoney & McEvoy, 2012; McEvoy & Mahoney, 2012), including cyberchondria (possibly due to its relationship with anxiety and depression Carleton, 2016; Norr et al., 2015). Cyberchondria may develop through IU when a person with difficulties in facing uncertainty endeavors to reduce the uncertainty about symptoms of physical illness. A search for health information online may serve as a reassurance seeking, i.e., an attempt to decrease anxiety, which, if it fails, leads to even greater anxiety and further searching (Fergus, 2013; Norr et al., 2015).

Individuals with higher IU would be more motivated to engage in smartphone use to reduce distress, putting themselves at risk of developing PSU/SA. A similar process was described in the context of distress (in)tolerance (see Elhai et al., 2018). We posit that the motivation to use smartphones in people with increased IU and cyberchondria is analogous but with one specific difference. That is, in the context of both elevated IU and cyberchondria, people might use smartphones as a means to reassure themselves, i.e., to decrease anxiety by resolving uncertainty. However, in the context of trait IU, the uncertainty could be related to any life circumstances. In contrast, in the context of cyberchondria, this uncertainty is more specifically related to health symptoms. Therefore, we wanted to examine whether those specificities related to cyberchondria have an incremental predictive power when predicting SA, compared to the IU.

Also, since it has been shown that IU was related to externalizing symptoms, it is plausible to assume that it will be associated with SA. Lastly, we expect a moderate positive relationship between depression, anxiety symptoms, and SA, with their effect decreasing after including IU and cyberchondria in the model (Elhai et al., 2017a, 2017b), after accounting for age, gender, the purpose of smartphone use, and average daily smartphone use.

## Aim and Hypotheses

The present study aimed to further investigate the IU and cyberchondria as two potentially important constructs in explaining SA. Our main hypotheses were that cyberchondria will have a unique contribution to predicting SA, in a positive direction after IU is already included in the model since it has been suggested that cyberchondria is a distinct construct from other closely related constructs and it is related to PIU and SA/PSU (Köse & Murat, 2021; Starcevic et al., 2019). In addition, since the IU is a trait-like construct (Carleton, 2016), unlike cyberchondria, a potential nosological category, the IU was entered into the model first. Next, we expected the IU to be positively related to SA (Rozgonjuk et al., 2019), over and above depression and anxiety symptoms.

## Method

### Participants

This study used data gathered in another study that dealt with the Serbian adaptation of the Smartphone Application-Based Addiction Scale (Csibi et al., 2018; Vujić et al., 2023), where a convenience sample was collected online by sharing the link to the questionnaire on various social networks and messaging applications. Participants needed to be over 18 years old, smartphone users, and fluent in Serbian. In this study, the responses from people with a chronic disease (*n* = 128) were filtered out since the target population had to be chronic disease free. Therefore, this study is based on 471 participants, 260 women (55.2%) and 211 men (44.8%). The average age of the participants was *M* = 38.67 (*SD* = 10.41). Most participants had finished college or university (41.2%), 23.8% had master’s degrees, 5.31% had a Ph.D. or higher, and 22.5% graduated from high school. Two participants had primary school education, while 6.79% were studying at the university at the time of data collection. The participants rated their financial situation as follows: 7.22% as very good, 36.70% as good, 47.60% as average, 7.43% as poor, and 1.06% as very poor.

### Materials

#### Demographic Questions

Participants were asked about their gender, age, level of education, and current financial status.

#### Questions About the Daily Use of Smartphones

Participants were required to estimate their daily smartphone (SP) use in hours on a typical workday and weekend. First, a unique variable of smartphone use was computed by multiplying typical daily use over workdays by five and multiplying the use over the weekend by two. Then the sum of the two was divided by seven.

In the case of extreme responses (two in the dataset), the highest value of the weekday and weekend use was substituted with the next highest value plus one before computing the mean, as Field et al. (2012) suggested. Namely, an extreme value of 24-h use on weekends was replaced by 21 (the next highest value plus one), and similarly, 23-h use on workdays was replaced by 17. We believe that the transformation was justified since we found it unrealistic that someone uses a smartphone 24 or 23 h a day. Yet, we avoided deleting the extreme values because those answers reflect the participants’ very high daily smartphone use.

#### Smartphone Use Purpose Questions

Two questions were used to assess two broad purposes of smartphone use, namely entertainment/leisure and productive use. The first was “How often do you use your smartphone for fun, out of boredom or habit (e.g., watching videos, scrolling through social media, listening to music, surfing the internet, etc.)?” and the second was “How often do you use your smartphone to fulfill a certain task (e.g., communicating with friends and family, paying bills, navigation, using a smartphone for work or for study purposes.” Both questions were answered on a 7-point Likert scale, from 1 = *almost never* to 7 = *almost always*.

#### Smartphone Application-Based Addiction Scale (SABAS; Csibi et al., 2018; Validation Study of the Serbian Translation of the Scale Can Be Found in Vujić et al., 2023).

This is a 6-item scale based on a components model of addiction (Griffiths, 2005). Each item refers to one of the components; however, the total score is used to operationalize smartphone addiction. The response scale is Likert type (1 = *strongly disagree* to 6 = *strongly agree*). The reliability of the instrument in the form of Cronbach’s *α*, and McDonald’s *ω* total in this study was *α* = 0.82 (*ω* = 0.82), practically the same as in the English version evaluation study, where it was *α* = 0.81 (Csibi et al., 2018).

#### Short Cyberchondria Scale (SCS; Jokić-Begić et al., 2019)

This brief scale is constructed to measure the core features of cyberchondria. Sample items include “After searching for health information, I feel frightened,” and “Once I start searching for health information, I find it difficult to stop.” It was previously adapted from the Croatian language and validated into Serbian (Vujić et al., 2022) but was also used in English (Farooq et al., 2020). It is a reliable scale with good psychometric characteristics. The respondents answer on a 5-point scale (1 = *I totally disagree*, 5 = *I totally agree*). The reliability of the scale in this study was *α* = 0.86 (ω = 0.86), similar to another study, where it was *α* = 0.82 (Vujić et al., 2022).

#### Intolerance of Uncertainty Scale (IUS-11; Mihić et al., 2014)

This short version was constructed directly from the full English version of IUS (Freeston et al., 1994), and not by translating the short English version, which contains 12 items (Carleton et al., 2007). For the Serbian language, the 11 items appeared to be optimal. Sample items include “When it’s time to act, uncertainty paralyzes me” and “One should always look ahead to avoid surprises.” The instrument showed good psychometric properties (Mihić et al., 2014). It can measure two dimensions of IU, inhibitory anxiety (IA, “Uncertainty makes my life intolerable”) and prospective anxiety (PA, “Unforeseen events upset me greatly”); however, the total score can be computed to represent general IU (Mihić et al., 2014; Renjan, 2016). The response format is a 5-point Likert (1 = *not at all characteristic of me*, 5 = *entirely characteristic of me*). In this study, the total score was used. The internal consistency of the whole scale was *α* = 0.91 (*ω* = 0.91) in this study.

#### Depression Anxiety Stress Scale (DASS-21;Jovanović et al., 2014; Lovibond & Lovibond, 1995)

The DASS-21 depression and anxiety scales were used, each containing seven items with the 4-point Likert response scale (0 = *did not apply to me at all*, 3 = *applied to me very much or most of the time*), asking participants to rate their feelings in the past week. The depression scale depicts depressive symptoms such as anhedonia and dysphoria, while the anxiety scale portrays anxious symptoms defined as arousal, physical symptoms, and subjective uneasiness. In the current work, Cronbach’s alpha of the depression scale was *α* = 0.86 (*ω* = 0.87), and anxiety was *α* = 0.80 (*ω* = 0.81).

### Procedure

Data were collected online, using the Qualtrics platform, for the study on adapting the Serbian version of SABAS, at the beginning of 2022 (see Vujić et al., 2023). The original study from which these data came from obtained ethical approval from the Research Ethics Board of the first author’s university (2020/306).

### Data Analysis

In the correlational analysis, we used the Pearson correlation coefficient, where the *p*-values were corrected via the Benjamini–Hochberg method, controlling the false discovery rate, in our case, the rate of 0.05 (Benjamini & Hochberg, 1995). Therefore, all adjusted *p*-values below 0.05 were considered significant. Next, we used hierarchical multiple regression analysis, with smartphone addiction as the outcome variable. A composite score was computed using depression and anxiety symptoms scales from the DASS-21 questionnaire by taking the mean of the scores of the two scales. That was done due to their substantially high correlation to avoid potential multicollinearity problems in the regression model. Furthermore, calculating the total DASS-21 score is possible, representing general distress. Since we had not administered the stress scale, we decided to make a composite score with only anxiety and depression. The composite was named *depression and anxiety symptoms*.

There were five regression steps, and the authors determined the order of the inclusion of the variables. In the first step, gender and age were entered. The second step included entertainment and productive use frequency, as well as the average duration of smartphone use. In the third step, the composite score of the depression and anxiety scale was entered. In the fourth step, intolerance of uncertainty was added, and in the fifth step, cyberchondria was added, making it eight predictors overall. Data were analyzed using the R programming language (R Core Team, 2022), including “tidyverse” (Wickham et al., 2019), “psych” (Revelle, 2022),“sjPlot” (Lüdecke, 2021), “lm.beta” (Behrendt, 2023), “haven” (Wickham et al., 2022), “broom” (Robinson et al., 2023), “dlookr” (Ryu, 2022), “janitor” (Firke, 2023), “car” (Fox & Weisberg, 2019), “MASS” (Venables & Ripley, 2002), “robustbase” (Maechler et al., 2022), and “lmtest” (Zeileis & Torsten, 2002) packages.

## Results

### Descriptive Statistics

Descriptive statistics are shown in Table 1. It should be noted that daily smartphone use in hours and depression had notably skewed distributions. Since the sample was taken from the general population and with people suffering from chronic diseases excluded, it was expected that most depression and anxiety symptoms scores would be low.

**Table 1:** Descriptive statistics

Variable	*M*	*SD*	*IQR*	*Skew*	*Kurt*	*Min*	*Mdn*	*Max*
Smartphone addiction	15.69	5.74	8	0.32	− 0.50	6	15	33
Entertainment use	5.05	1.39	2	− 0.34	− 0.41	1	5	7
Productive use	5.77	1.34	2	− 1.08	0.58	1	6	7
Average SP use per day (h)	4.01	2.37	2.43	1.62	3.79	0.5	3.57	17.14
Anxiety	2.59	2.88	4	1.69	3.86	0	2	20
Depression	3.12	3.54	3.50	1.71	3.29	0	2	20
DA symptoms	2.86	2.93	3.50	1.76	3.79	0	2	18
Intolerance of uncertainty	23.34	8.00	11	0.88	0.44	11	22	52
Cyberchondria	8.42	3.83	6	0.73	− 0.08	4	8	20

*M*, mean; *SD*, standard deviation; *IQR*, interquartile range; *Skew*, skewness; *Kurt*, kurtosis; *Min*, minimum; *Mdn*, median; *Max*, maximum; *DA symptoms*, depression and anxiety symptoms composite

Table 2 shows the Pearson correlation between the variables. Smartphone addiction had the highest correlation with entertainment use purpose, followed by correlations with intolerance of uncertainty, daily smartphone use, and cyberchondria. These correlations were moderate and positive.

**Table 2:** Pearson correlation coefficients

Variable		1	2	3	4	5	6	7
1	Age	1						
2	Smartphone addiction	− .11^*^	1					
3	Entertainment use	− .26^***^	.55^***^	1				
4	Productive use	.01	.20^***^	.38^***^	1			
5	Average SP use per day (h)	− .18^***^	.35^***^	.48^***^	.22^***^	1		
6	DA symptoms	− .15^**^	.30^***^	.21^***^	− .02	.16^**^	1	
7	Intolerance of uncertainty	− .01	.34^***^	.19^***^	.10^*^	.08	.49^***^	.1
8	Cyberchondria	− .16^**^	.31^***^	.17^***^	− .02	.14^**^	.38^***^	.38^***^

The *p*-values were adjusted for multiple tests using the Benjamini–Hochberg method; *DA symptoms*, depression and anxiety symptoms composite

^*^p < .05, ^**^p < .01, ^***^p < .001

### Regression Analysis

Inspection of the *VIF* values indicated no danger of multicollinearity in the hierarchical regression since all were < 2, while usually, *VIF* < 5 is considered problematic (Sheather, 2009). Visual inspection of the final model residual distribution indicates no violation of normality assumptions. No cases with the Cook’s distance greater than one would be considered highly influential (Field et al., 2012). We should note that due to a possible slight violation of the homoscedasticity assumption and 11 multivariate outliers (detected via Mahalanobis distance and studentized residuals), we decided to run a robust regression model and a model with the heteroscedasticity consistent standard errors, with all predictors, included. However, no substantial differences in estimates, standard errors, and corresponding *t*-values were found in comparison to the regular regression model. Namely, the predictors that were significant at 0.05 level remained significant in both the robust model and the model with adjusted standard errors. Therefore, the same conclusion could be drawn from robust and non-robust models, indicating that the mentioned issues did not notably bias the results.

All five models had a significant *F*-value, and each subsequent step significantly improved the model. This finding indicates that each block of variables substantially improved the explanation of smartphone addiction. Since intolerance of uncertainty and cyberchondria were entered individually at the fourth and fifth steps, both variables uniquely contributed to predicting smartphone addiction, over and above depression and anxiety symptoms, and while controlling for daily smartphone use, the purpose of use, gender, and age. The cyberchondria that was included as the last predictor had a somewhat weaker effect than IU. According to standardized coefficients, the effects of IU and cyberchondria could be described as minor. The final model explained nearly 40% of the outcome variance. In the final model, significant predictors were entertainment use, intolerance of uncertainty, cyberchondria, and the duration of daily use of smartphones, all predicting smartphone addiction in a positive direction, as expected. Standardized estimates indicate that the effect of entertainment use was much more significant than the effects of IU and cyberchondria (Table 3).

**Table 3:** Results of hierarchical regression analysis with smartphone addiction as the outcome

Predictors	Model 1		Model 2		Model 3		Model 4		Model 5	
	*b*	*β*	*b*	*β*	*b*	*β*	*b*	*β*	*b*	*β*
Constant	17.58^***^	-	3.15^*^	-	2.25	-	0.43	-	− 0.86	-
Gender (female)	1.25^*^	.11	1.05^*^	.09	0.85	.07	0.89^*^	.08	0.75	.07
Age	− 0.07^**^	− .12	0.02	.04	0.03	.06	0.02	.04	0.03	.05
Entertainment use			2.12^***^	.51	1.97^***^	.48	1.88^***^	.45	1.86^***^	.45
Productive use			− 0.13	− .03	− 0.04	− .01	− 0.11	− .03	− 0.07	− .02
Average SP use per day (h)			0.30^**^	.12	0.27^*^	.11	0.29^**^	.12	0.27^**^	.11
DA symptoms					0.36^***^	.18	0.16	.08	0.10	.05
Intolerance of uncertainty							0.15^***^	.21	0.12^***^	.17
Cyberchondria									0.21^***^	.14
*R*^*2*^/adj. *R*^*2*^	.024/.020		.325/.318		.355/.347		.387/.378		.403/.392	
Δ*F*	5.84^***^		77.45^***^		23.68^***^		24.46^***^		12.03^***^	

*b*, unstandardized coefficient; *β*, standardized coefficient; *adj. R*^*2*^, adjusted squared multiple correlation; *ΔF*, change in the *F* statistic; *DA symptoms*, depression and anxiety symptoms composite

^*^p < .05, ^**^p < .01, ^***^p < .001

In the final model, gender was no longer a significant predictor, although its *p*-value was close to the threshold, *p* = 0.07, indicating somewhat higher scores on smartphone addiction in women than in men. We note that the mean score on SABAS for women was *M* = 16.20 (*SD* = 5.56), and for men *M* = 15.06 (*SD* = 5.90).

**Fig. 1:**
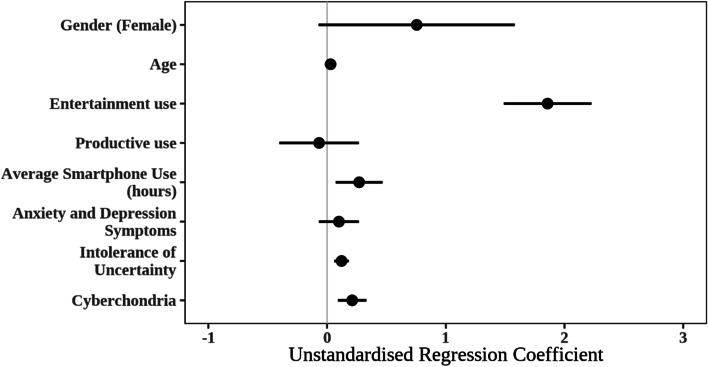
The model 5 regression estimates plot with the smartphone addiction as the outcome. *Note:* Error bars are 95% confidence intervals

The use of smartphones for productive purposes was not significant, despite its moderate and positive correlation with SA. Depression and anxiety symptoms were no longer significant after entering intolerance of uncertainty in the model. In summary, the model explained a substantial proportion of variance of smartphone addiction, with entertainment use, IU, cyberchondria, and smartphone daily use duration being significant predictors. Model 5 regression estimates with 95% confidence intervals are presented in Fig. 1 in the Appendix.

## Discussion

This study investigated the relationship between cyberchondria, IU, and PSU. Hierarchical linear regression results suggest that cyberchondria uniquely affects PSU, over and above anxiety and depression symptoms, and IU, with age, gender, purpose (entertainment use and productivity use), and duration of smartphone use accounted for. The results supported our hypotheses that both IU and cyberchondria will have a positive relationship with SA and that each will contribute uniquely to explaining SA. That is, people with higher IU, as well as cyberchondria, may be more likely to engage in PSU and potentially develop SA. However, concluding causal relationships would require a different longitudinal or experimental study design rather than a cross-sectional one. We should also note that a bidirectional relationship between the investigated constructs is possible. These findings align with previous research, which suggested the connection between IU (Rozgonjuk et al., 2019) and cyberchondria (Köse & Murat, 2021) on the one hand and SA on the other. It is also worth mentioning that the strongest predictor of SA in the model was entertainment use purpose, while productivity use purpose was not significant. This finding also agrees with previous research reports (van Deursen et al., 2015; Wang et al., 2015).

The relationship between IU and SA suggests that IU is a core aspect of different psychopathologies or maladaptive behaviors (Carleton, 2016; Carleton et al., 2019; Mahoney & McEvoy, 2012; McEvoy & Mahoney, 2012). Intolerance of uncertainty can promote maladaptive coping in the shape of SA (Rozgonjuk et al., 2019) because some people tend to resolve the uncertainty and reduce distress related to it by any means (e.g., reassurance-seeking behaviors or excessive collecting of information). However, increased smartphone use could actually produce the opposite effects, leading to higher IU. Smartphones provide the possibility of constant reassurance (e.g., that a family member arrived home safely; that the partner is not cheating, etc.), but the perpetual availability of “safety cues” eventually can increase anxiety and deprive distress tolerance, leading to the rise of perceived uncertainty (Carleton et al., 2019). In other words, the maladaptive coping reflected in smartphone overuse could possibly maintain the aversive emotions. Most activities performed using a smartphone cannot actually reduce the uncertainty and negative emotions accompanied by it, no matter how much information a person gathers. However, this coping strategy can bring some temporary relief in some situations, which only reinforces this dysfunctional coping strategy, bringing negative outcomes in the long run. These negative outcomes are inevitable because reassurance-seeking as a safety behavior prevents people from testing their irrational beliefs about uncertainty in reality and from learning to tolerate uncertainty (Carleton et al., 2019).

Aside from reassurance-seeking, avoiding uncertainty-related negative feelings is the other way to cope with uncertainty-related negative feelings. The usage of smartphones for both reassurance-seeking and avoiding facing uncertainty can be explained from the compensatory internet use theory perspective. According to the theory, the internet, or smartphone platforms, can be used as a coping strategy with negative outcomes. In other words, activities such as playing games or scrolling through social media can be used to escape real-life problems or  reduce stress (Kardefelt-Winther, 2014). For example, individuals who use smartphones as a coping tool can increase entertainment activities on their devices when they want to distract themselves from negative emotions and ongoing real-life problems (Wang et al., 2015). A practical implication of the findings is that if one of the primary motivations of an individual to overuse, a smartphone is the reduction of uncertainty, then an intervention aiming to increase the ability to tolerate uncertainty and decrease biased interpretation of uncertainties could be implemented to help a person deal with the PSU/SA (see Oglesby et al., 2017). This intervention could also be a treatment of choice based on our finding that symptoms of depression and anxiety were no longer significant after including IU in the model but also based on the existing knowledge that IU underpins various emotional and behavioral problems (Mahoney & McEvoy, 2012; McEvoy & Mahoney, 2012). Finally, Qiu and colleagues (Qiu et al., 2023) have also recently recognized that targeting IU in therapy could be a preventive measure for PSU/SA.

Results also suggested that, despite both IU and cyberchondria having a similar proposed underlying motivation for smartphone use, namely, reducing the uncertainty, the cyberchondria had a unique contribution to predicting SA, above and beyond IU. The uncertainty in the context of the IU could be related to any aspect of life. In contrast, in the context of cyberchondria, uncertainty is specifically related to health symptoms, which a person tends to reduce. Additionally, in cyberchondria, the tendency to reduce uncertainty and self-reassure includes, by definition, searching for health information online, which can be performed via smartphone (among other devices). Accordingly, the behavioral aspect of the connection between cyberchondria and SA is more obvious than it is in the case of the IU and SA relationship, where IU can have various behavioral expressions, with smartphone overuse/SA being only one of them.

Nevertheless, a smartphone is only an instrument or a medium through which certain activity is performed, and the results must be interpreted with this in mind. The excessive use of smartphones, and therefore SA, may reflect an underlying compulsion (for example, online shopping, gaming, watching videos, scrolling through social media) which serves as a way of dealing with distress and anxiety. A previous study showed that individuals “deliberately engage in specific activities with specific content…” (Pontes et al., 2015, p. 23), so if they were unable to access their chosen activities, they would stop going online completely, or they would substantially decrease their time spent online (Pontes et al., 2015).

The average time participants spent using the smartphone during the day is quite similar to what was reported in other studies, including the Serbian one (Kwon et al., 2013; Nikolic et al., 2022). Contrary to what was hypothesized, depression and anxiety symptoms were not significant predictors of SA (although the *p*-value was close to the threshold of 0.05), after including IU in the model despite DA symptoms being moderately correlated with SA. The nonsignificance of DA symptoms as predictors might be due to the substantial overlapping of the variance in DA symptoms and IU, which explains the SA variance. The insignificant effect of age is consistent with some previous studies (Elhai et al., 2017a, 2017b; Kuss et al., 2018; Moreno-Guerrero et al., 2020) but also in contradiction with others (Csibi et al., 2021; De-Sola Gutiérrez et al., 2016; Mitchell & Hussain, 2018; van Deursen et al., 2015). The relation between age and SA seems highly dependent on the sample, consisting of high school or university students, participants from the general population with different age ranges, etc. Another possible reason for the non-significant effect of age could be the non-linear relation between age and SA, for which a linear model would not be appropriate. However, the investigation of this relationship was not the focus of this report. Some studies showed greater problematic smartphone use in women (De-Sola Gutiérrez et al., 2016; Lopez-Fernandez et al., 2017; van Deursen et al., 2015), while others did not show gender differences (Mitchell & Hussain, 2018). It is also possible that the sample age structure plays a role in these diffusing findings.

## Limitations and Future Directions

The first limitation of the study is its cross-sectional design, which does not allow us to conclude anything about causality in the relationship between IU, cyberchondria, and SA. Therefore, future studies should consider a longitudinal design to overcome this shortcoming. Second, a convenient online sample was tested in this study, and it was not representative of the Serbian general population since the self-selection bias in this sample could be present. Therefore, subsequent studies should use random sampling to collect a more representative sample. Furthermore, to isolate the potential unique variance SA in its relationship with IU and cyberchondria, a measure of PIU (or IA) should be included in the model, which has not been done in this study, to have a more detailed insight into whether the smartphone as a medium facilitates cyberchondria. Next, a self-report of smartphone screen time could be biased; thus, a more objective measure could be a better alternative in assessing daily smartphone use duration (for example, using a smartphone application for obtain the usage data or using the data provided by the smartphone operating system). Finally, the study is based on a Serbian sample which means its cross-cultural generalizability is questionable. Despite these limitations, we hope this research will encourage further investigation of the problematic use of technology and cyberchondria, its diathesis, and connection with psychopathological consequences.

## Data Availability

The data and the R code are available upon request from the first author (vujic.aleksandar@ppk.elte.hu).

## Appendix

1
